# T cell-mediated immune responses in xenotransplantation: mechanisms and therapeutic strategies

**DOI:** 10.3389/fimmu.2026.1767840

**Published:** 2026-02-23

**Authors:** Ziyue Wang, Guomu Liu

**Affiliations:** 1 Key Laboratory of Organ Regeneration & Transplantation of Ministry of Education, The First Hospital of Jilin University, Changchun, China; 2 Department of Dermatology and Venereology, The First Hospital of Jilin University, Changchun, China

**Keywords:** immunological tolerance, immunosuppression, T cell-mediated rejection, xenoantigen, xenotransplantation

## Abstract

Xenotransplantation has emerged as a promising approach to alleviate the shortage of donor organs. While advances in porcine gene-editing technologies have largely overcome hyperacute rejection, the persistent challenge of T cell- and antibody-mediated rejection reactions continues to impede the long-term survival and clinical translation of xenografts. T cells connect innate and adaptive immunity and are the main effector cells in cell-mediated rejection reactions. The antigen recognition, activation, and effector differentiation of T cells have a crucial impact on the clinical outcomes of xenotransplantation. This review focuses on the immune response process of T cells in xenotransplantation and discusses potential strategies for T cell-mediated rejection reactions. Future exploration of efficient and safe approaches for inducing immune tolerance will be a key direction for prolonging the survival of xenografts.

## 1 Introduction

Transplantation remains one of the most effective therapies for end-stage organ failure (1, 2). However, the number of patients waiting for transplantation continues to rise, whereas the supply of suitable human donor organs is severely insufficient (3–5). Xenotransplantation is a promising strategy to alleviate this shortage. Pigs are regarded as particularly suitable donors due to multiple advantages (6). Pigs share similarities with humans in physiology and anatomy; moreover, they can grow rapidly and be raised on a large scale, making them an “off-the-shelf” universal product. Most importantly, pig gene editing technology is mature, allowing for multiple edits (7, 8). Additionally, the ethical considerations of using pigs as donors are more favorable compared to other species (5, 9). In recent years, the field of xenotransplantation has made rapid progress through the optimization of multi-gene editing strategies in donor pigs and immunosuppressive regimens. Currently, gene editing strategies for donors typically involve knocking out major xenogeneic carbohydrate antigens (GGTA1, CMAH, B4GALNT2), while introducing human complement regulatory proteins (hDAF, hCD46, hCD59), coagulation regulatory molecules (hTBM, hEPCR), and factors regulating innate immunity and inflammation (hCD47, HO-1), thereby obtaining xenografts with low immunogenicity and high physiological compatibility (10–12). On the recipient side, immunosuppressive regimens centered on anti-CD40 or anti-CD154 monoclonal antibodies have further reduced early antibody- and T cell-mediated damage. Based on these advancements, non-human primate (NHP) models have achieved unprecedented xenotransplant survival. The longest survival time of heterotopic heart transplants reached 945 days (13), that of orthotopic heart transplants reached 9 months (14), and that of kidney transplants reached 758 days (15). Publicly reported follow-up has reached 271 days of xenokidney function, detailed peer-reviewed clinical reporting is still emerging (16, 17).

Despite the advancements, T cell- and antibody-mediated rejection continues to hinder the clinical application of xenotransplantation (18, 19). This suggests that when hyperacute rejection is effectively managed, adaptive immune responses will become the main factor in xenotransplant outcomes (18, 19). T cells play a core role in this process. As a key bridge between innate and adaptive immunity, conventional αβ T cells cannot only directly attack the graft but also promote antibody responses as well as the recruitment and activation of innate cytotoxic cells via cytokine production. Compared with the limited HLA mismatch in allogeneic transplantation, the extensive species antigenic differences between humans and pigs make the T cell response in xenotransplantation more complex and intense (19–21). Human αβ T cells respond to porcine aortic endothelial cells (PAECs) exhibit more vigorous cell proliferation and cytokine production than they do to human umbilical vein endothelial cells (HUVECs) (22). It has been reported that approximately 0.1% to 10% of peripheral αβ T cells are alloreactive (23–25). Notably, the frequency of xenogeneic donor-reactive T cell clones (XDRTCCs) before transplantation was less than 0.1%, but after kidney xenotransplantation, XDRTCCs rapidly expanded in the peripheral blood (26). Although T cells are typically classified as a component of the adaptive immune response, γδ T cells exhibit characteristics of both innate and adaptive immunity (27, 28). In xenotransplantation, it is evident that certain γδ T cell TCR clones experience substantial clonal expansion (29). These results suggest that although xenotransplantation shares many molecular features with allografts, the resulting T-cell response is usually more intense and develops more rapidly.

This review not only summarizes the current state of scientific research on T-cell immune responses in xenotransplantation but also delves into key strategies aimed at overcoming these immunological challenges.

## 2 Mechanisms of T cell recognition

In xenotransplantation, T cell-mediated rejection reactions require recipient T cells to recognize porcine xenogenic antigens. Understanding these immunological mechanisms is crucial for developing of gene editing strategies for donor pigs and advancing therapeutic drugs aimed at mitigating rejection. Below, we will discuss research advances in understanding the T cell recognition mechanism in xenotransplantation.

### 2.1 Direct pathway

**Figure 1:**
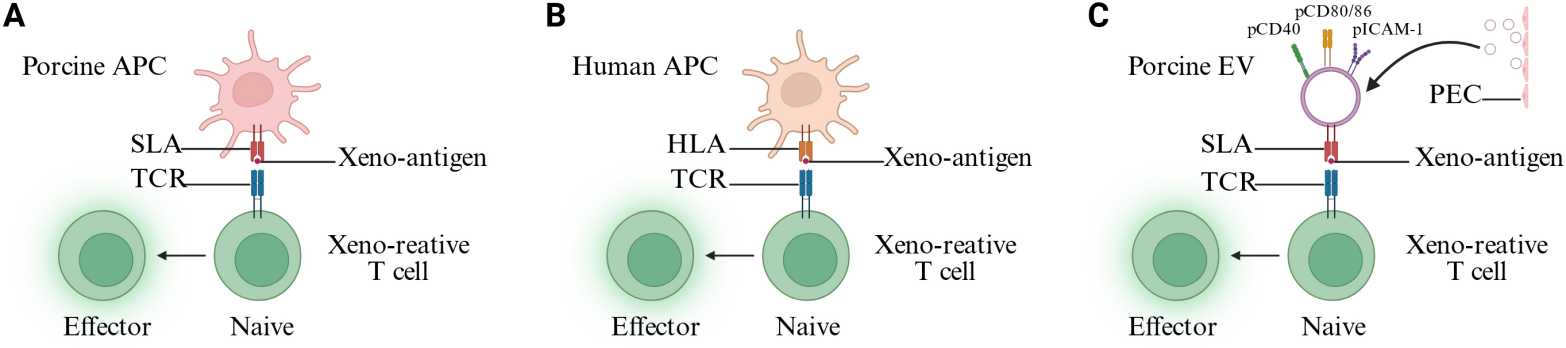
Mechanisms of xenorecognition at the priming stage (Created with BioRender). **(A)** Pig APCs carry out the initial activation of xenoreactive T cells by presenting complete donor antigen–SLA complexes to human T cells (direct pathway). **(B)** Human APCs carry out the initial activation of xenoreactive T cells by presenting complete donor antigen–HLA complexes to human T cells (indirect pathway). **(C)** PECs secrete EVs that express SLA. These EVs can directly present antigen-SLA complexes to human T cells in an APC-independent manner (semi-direct pathway). APCs, antigen-presenting cells; SLA, swine leukocyte antigen; TCR, T cell receptor; HLA, human leukocyte antigen; PECs, porcine vascular endothelial cells; EVs, extracellular vesicles.

Human T cell receptors (TCRs) are capable of binding to swine leukocyte antigen (SLA) I and II molecules on professional antigen-presenting cells or non-professional antigen-presenting cells of pigs (30–32) (**Figure 1A**). Donor passenger leukocytes are rapidly cleared after xenotransplantation—largely through macrophage-mediated innate immunity (for example, owing to CD47–SIRPα incompatibility)—thereby confining direct T-cell priming to the early post-transplant period and promoting a subsequent shift toward indirect and semi-direct pathways (21, 33–35). Nevertheless, graft vascular endothelial cells persist and can act as non-professional antigen-presenting cells, supporting local antigen presentation and episodic re-stimulation of infiltrating T cells. In a 61-day pig-to-human decedent model, combined direct and indirect mixed lymphocyte reaction (MLR) were used to define XDRTCCs. Across all time points, a substantial fraction of detected CD4+ XDRTCCs were directly xenoreactive, whereas a smaller fraction showed indirect reactivity; notably, the proportion of directly xenoreactive CD8+ XDRTCCs increased from 15% before transplantation to 73% by postoperative day 49 (26). In a porcine lung allotransplantation model (36), lentiviral vectors targeting B2M and CIITA through ex vivo lung perfusion significantly downregulated the expression of SLA-I/II in porcine lung grafts and achieved long-term survival without immunosuppression. In xenotransplantation, targeting B2M and CIITA in donor pigs by CRISPR/Cas9 and other methods can almost completely eliminate the expression of SLA-I/II; however, this alone is insufficient to induce stable immune tolerance. Unlike allotransplantation, in which antigenic disparity is largely defined by limited MHC mismatching, SLA constitutes only a subset of xenoantigens. Consequently, although targeted deletion of B2M and CIITA can markedly attenuate or even abrogate direct antigen presentation, recipient T cells can still be activated via indirect recognition and thereby mediate rejection (37–39).

### 2.2 Indirect pathway

The indirect antigen recognition pathway in xenotransplantation is essentially the same as that in allogeneic transplantation (**Figure 1B**). Both involve the processing of donor antigens by the recipient antigen-presenting cells (APCs) and their presentation to T cells via the recipient MHC (40, 41). In xenotransplantation, the indirect recognition faces a highly heterogeneous xeno-peptide library. Studies have shown that the indirect pathway is more advantageous in triggering xenogeneic reactions than allogeneic reactions (42). Bühler group directly quantified this in the pig cell/organ to baboon model using ELISPOT. The direct T cell xenoreaction diminished rapidly over time, whereas the CD4+ T cells response activated by the indirect pathway could be detected for a long time and was associated with pathological damage to the graft (21).

### 2.3 Semi-direct pathway

Previous studies have demonstrated that the APCs of the donor can release extracellular vesicles (EVs) (43–45). These EVs carry antigen-MHC complexes, co-stimulatory proteins, and adhesion molecules (44, 46). Once the recipient APCs obtain these extracellular vesicles, they can present them as intact molecules to the T cells (46, 47). This pathway is called the semi-direct antigen presentation pathway. When vascular injury, rejection, or thrombosis occurs, human endothelial cells release EVs. These EVs account for 15% of circulating EVs (48). Porcine vascular endothelial cells (PECs) can release extracellular vesicles expressing SLA-I and SLA-DR (49). However, only EVs expressing SLA-I can bind to human CD14+, CD4+, and CD8+ cells. Unlike the traditional semi-direct antigen recognition pathway, although CD14+ monocytes can be modified by EVs expressing SLA-I, these EV-pulsed CD14+ monocytes cannot promote T cell proliferation (49). These endothelium-derived EVs directly initiate the immune response of recipient T cells through SLA-peptide complexes and co-stimulatory molecules. This non-traditional semi-direct pathway is also referred to as the “secondary-direct” pathway (**Figure 1C**). Reducing or eliminating the expression of SLA may be a method to alleviate the immune response induced by extracellular vesicles (49).

## 3 T-cell co-stimulatory and co-inhibitory pathways

**Figure 2:**
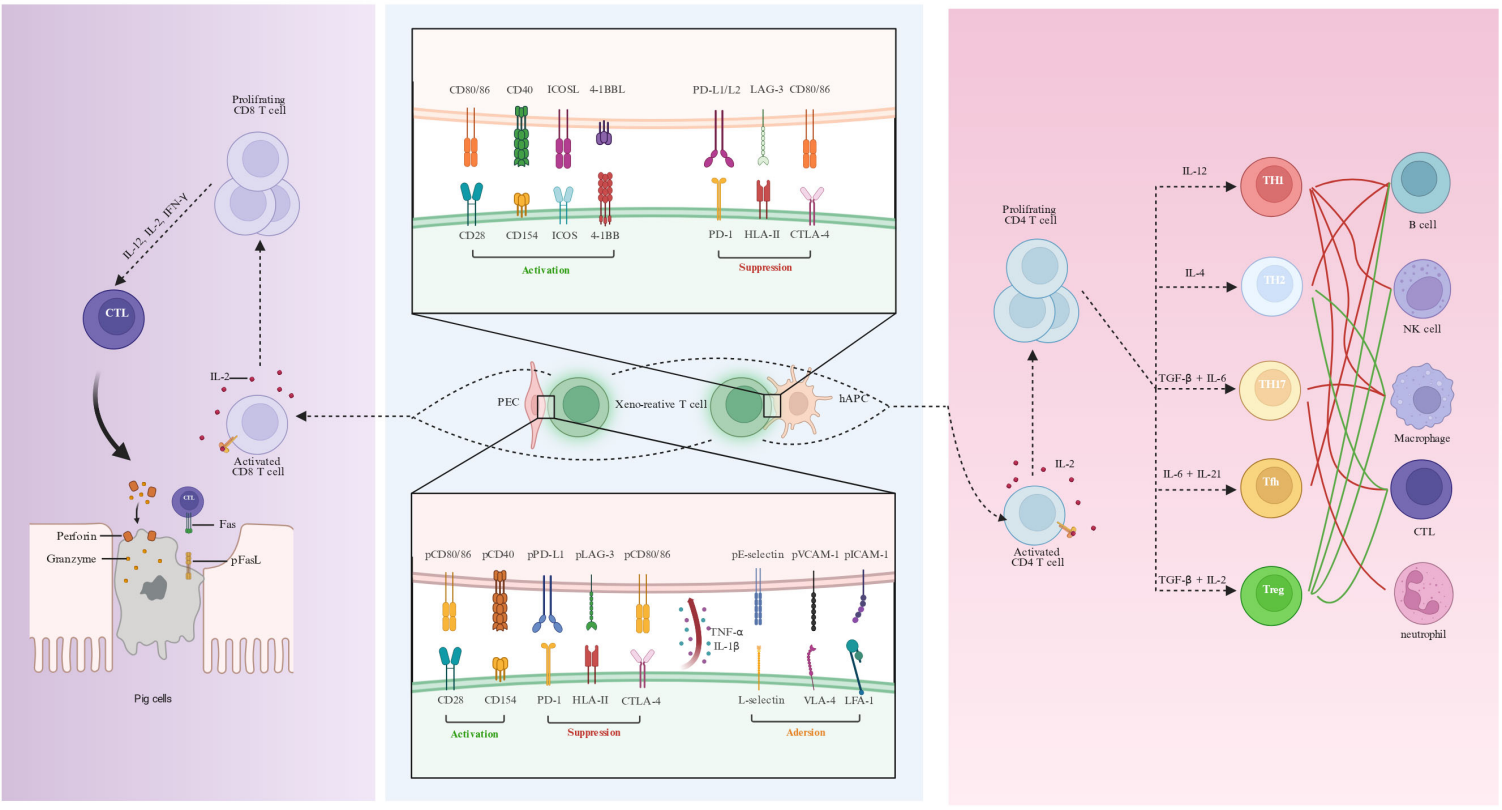
T cell co-stimulatory pathways, subset differentiation, and effector mechanisms in xenotransplantation. Multiple co-stimulatory ligands present on porcine endothelial cells bind cross-species to their corresponding receptors on human T cells. T cells activated via the pCD80/CD86–CD28 and pCD40–CD154 pathways generate inflammatory cytokines, such as TNF-α and IL-1β; these cytokines upregulate endothelial adhesion molecules (E-selectin, VCAM-1, and ICAM-1) and promote the recruitment of human T cells through L-selectin, VLA-4, and LFA-1, thereby facilitating the rejection reaction. Meanwhile, co-inhibitory pathways including pPD-L1–PD-1, pLAG-3–HLA-II, and pCD80/CD86–CTLA-4 also display cross-species reactivity. T cells activated through the indirect antigen recognition pathway possess co-stimulatory and co-inhibitory pathways similar to those in allografts in terms of molecular composition, such as CD40-CD154 and CD80/CD86-CD28/CTLA-4; however, they exhibit unique characteristics in terms of reaction intensity specific to xenotransplantation. Distinct THs assume a central regulatory function in adaptive immune responses through the secretion of cytokines and the provision of co-stimulatory signals. These signals coordinate and augment the differentiation and function of diverse immune cells, including B cells, phagocytes, neutrophils, NK cells, and CTLs. CTLs induce apoptosis of xeno-graft cells through perforin/granzyme and Fas/FasL pathways and are the principal effector cells in cell-mediated rejection. hAPC, human antigen-presenting cell; PEC, porcine vascular endothelial cell; CTLs, cytotoxic T lymphocytes.

After T cells recognize porcine xenogenic antigens via the direct antigen recognition pathway, porcine co-stimulatory molecules are still capable of cross-species interaction with the corresponding receptors on the surface of human T cells (**Figure 2**). In contrast to humans, porcine APCs, including dendritic cells and endothelial cells, are capable of continuously expressing pCD80/CD86 (50–53). pCD86 can promote the proliferation of human T cells and IL-2 secretion. Moreover, the co-stimulatory effect of pCD86 on human T cells can be blocked by the human CTLA4Ig fusion protein (52). The CD40-CD154 axis plays an important role in the interaction between T cells and APCs as well as the activation of vascular endothelial cells. Human CD154 can stimulate porcine endothelial cells to induce the expression of MCP-1, IL-8, IP-10, and RANTES (54). When human T cells are co-cultured with PECs, the inflammatory cytokines produced by the human T cells (especially TNF-α, IL-1β, but not IFN-γ) can directly act on PECs, upregulating SLA-I/II, E-selectin, VCAM-1, and ICAM-1 molecules. The expression of these porcine adhesion molecules can recruit human T cells through L-selectin, VLA-4, and LFA-1, thereby promoting the rejection reaction (55–58). Through sequence homology analysis and structural analysis, the structure level supports pCD58 as the functional ligand of human CD2. In terms of co-inhibitory molecules, the porcine PD-L1 has a 73.8% sequence homology with human PD-L1 and has a similar molecular structure. The porcine PD-L1 can inhibit the proliferation of human CD4+ T cells by anti-CD3 and anti-CD28 and promote their apoptosis (59). Soluble porcine LAG-3 can bind to porcine and human MHC II molecules, and has an inhibitory effect on the proliferation of human lymphocytes in the human-porcine mixed lymphocyte reaction (60). As the direct antigen recognition pathway diminishes, the APC of the human can still continuously uptake, process, and present xenogenic antigens (61). The T cells activated by the indirect antigen recognition pathway exhibit co-stimulatory and co-inhibitory pathways that are molecularly similar to those found in allogeneic transplantation (40). However, they exhibit characteristics specific to xenotransplantation in terms of response intensity (62). From the perspective of molecular pathways, the indirect pathway is mediated by a series of classical co-stimulatory molecules, such as CD80/CD86-CD28/CTLA-4, CD40-CD154, and ICOS-ICOSL (63). These signaling axes act in concert to drive the initiation and maintenance of T cell-dependent B-cell activation. Blocking the CD80/CD86-CD28 pathway can mitigate the antibody response against non-Gal antigens (64, 65). However, its effect on extending graft survival is relatively restricted. In contrast, blocking the CD40-CD154 pathway not only significantly suppresses T cell-mediated and antibody-mediated rejection but has also been demonstrated to be essential for attaining long-term survival in non-human primate xenotransplantation models (13–15, 66).

## 4 Crosstalk between T cells and other immune subsets

T cells serve as a crucial bridge connecting innate and adaptive immune responses in xenotransplantation and are the main executors of cellular immune rejection (34, 40). CD4+ T cells and CD8+ T cells jointly participate in xenograft damage. CD4+ T cells assist B cells in secreting xenospecific antibodies and activating the innate immune system; they also provide crucial auxiliary signals for the activation, clonal expansion, and differentiation into effector phenotypes of CD8+ cytotoxic T lymphocytes (CTLs) (67, 68). Meanwhile, effector CTLs act as the main executors of cell-mediated rejection, mediating the damage and destruction of the xenograft through direct cytotoxicity (67). Before T cell responses emerge, xenografts typically enter an early inflammatory phase characterized by endothelial activation, dysregulation of the complement and coagulation cascades, and the rapid sequestration of innate immune cells. Within hours to days post-transplantation, neutrophils, monocytes/macrophages, and natural killer (NK) cell are recruited to the graft (12, 69). Beyond mediating immediate tissue and microvascular injury, these innate populations drive T cell activation and effector differentiation by promoting antigen uptake and presentation and the release of pro-inflammatory cytokines such as IL-1β, TNF-α, IL-6, and IFN-γ (34, 70). Th1 secretes cytokines such as IFN-γ, which can promote NK cells activation, drive macrophage polarization to the M1 type, and support the differentiation and effector function of CTLs (56). In a humanized mouse model of xenogeneic islet transplantation, serum levels of IFN-γ, IL-6, and IL-17A increased, and Th17 cells were significantly infiltrated in the graft during the rejection period (71). Th17 recruits neutrophils by secreting IL-17A/F and cooperates to promote M1 polarization of macrophages, thereby amplifying local inflammation (72). Conversely, M1 macrophages secrete IL-12, IL-1β, TNF and IL-6, together with chemokines that bias T cell differentiation towards Th1 and Th17 lineages, establishing a feed-forward circuit that sustains effector T cell recruitment and tissue infiltration (73). In contrast, Th2 possibly participate in xenogeneic antibody-mediated rejection (AMR) and chronic injury by promoting B cell activation and antibody class switching (74). Regulatory T cells (Tregs) can mitigate the rejection response towards porcine islets in humanized mice and NHP, and prolong graft survival (75, 76). Large cohorts of allograft kidney transplantation studies have shown that the frequency and phenotype of T follicular helper cells (Tfh) cells are closely related to donor-specific antibodies (DSA) and AMR, suggesting that they also play an important auxiliary role in xenogeneic humoral immunity (77, 78). Meanwhile, B cells are not only effectors of humoral immunity but also function as highly efficient APCs and amplify inflammatory responses through antibody–complement pathways, thereby sustaining the activation of effector T cells (79, 80). CTLs directly cause graft cell death through cytotoxic effects mediated by granzyme/perforin, FasL-Fas-mediated apoptosis, and the release of inflammatory cytokines (81, 82). In a pig-to-macaque kidney transplantation model, selective depletion of CD4+ T cells rather than CD8+ T cells before transplantation enabled long-term survival, and only chronic antibody rejection symptoms appeared in the late stage. These findings suggest that immune regulation targeting CD4+ T cells or donor gene modification is a key strategy for prolonging graft survival and reducing rejection reactions (83).

## 5 The role of γδ T cells in xenotransplantation

γδ T cells exhibit both innate and adaptive immune characteristics. On the one hand, they express V(D)J rearranged γδ TCRs (29, 84). On the other hand, they co-express multiple NK cell-like activating and inhibitory receptors (85, 86). γδ T cells constitute approximately 1%–10% of CD3+ T cells in peripheral blood (87). Nevertheless, *in vitro* research has demonstrated that nearly 40% of human γδ T cell clones lyse PECs. This cytotoxicity mainly depends on the perforin–granzyme pathway driven by the γδ TCR–CD3 complex, rather than the Fas-FasL axis (88). In the pig to cynomolgus monkey vascular xenotransplantation model, the proportion of γδ T cells in peripheral blood steadily rises after transplantation, and a substantial number of γδ T cells can be observed infiltrating the graft, accompanied by the expansion of multiple clonal types predominantly dominated by δ1, δ3, or δ7 chains (29). Since γδ T cells are MHC-independent, they may have a role in humoral immunity that surpasses that of traditional αβ T cells, functioning as innate-like TFHs to facilitate the production of xenoreactive antibodies (89, 90). However, researchers inadequately understand the specific function of γδ T cells in xenotransplantation, necessitating more research to optimize immunosuppressive strategies and enhance transplant outcomes.

## 6 Multi-approaches to limit T cell xenoreactivity

Xenotransplantation, once a distant scientific aspiration, now stands on the brink of clinical reality, with numerous clinical reports documenting its progress (**Table 1**). The present research primarily focuses on overcoming the barriers to xenogeneic immunity and physiological compatibility to enhance graft stability and long-term survival. Gene editing technology is used to obtain xenogeneic grafts with low immunogenicity and high physiological compatibility (15). Immune suppression regimens centered on co-stimulation blockade effectively control rejection reactions (13). Building on these advancements, the pursuit of immune tolerance emerges as the ultimate goal, aiming to harmonize the recipient’s immune system with the xenograft. Thymus transplantation and mixed hematopoietic chimerism promote the establishment of central and peripheral tolerance by continuously presenting xenogeneic antigens in the thymus and periphery. Cell therapy enhances the persistence and stability of tolerance by strengthening peripheral immune regulation and antigen-specific inhibition.

**Table 1:** Current clinical experience with pig-to-human xenotransplantation.

Surgery date	Type of xenotransplantation	Genetic modifications	Patient status	Immunosuppression	Monitoring time	References
Sep. 2021	Thymokidney transplantation (Outside)	GGTA1 KO	Brain-dead	MMF+MP	54h	(69, 91)
NOV. 2021	Thymokidney transplantation (Outside)	GGTA1 KO	Brain-dead	MMF+MP	54h	
Sep. 2021	Kidney transplantation	4KO (GGTA1, CMAH, B4GALNT2, GHR) + 6KI (hDAF, hCD46, hTBM, hEPCR, hCD47, hHO1)	Brain-dead	rATG + Anti-CD20mAb(Rituximab) + TAC + MMF + MP	74h	(92–96)
–	Kidney transplantation	4KO (GGTA1, CMAH, B4GALNT2, GHR) + 6KI (hDAF, hCD46, hTBM, hEPCR, hCD47, hHO1)	Brain-dead	rATG + Anti-CD20mAb(Rituximab) + TAC + MMF + MP/Pred + Anti-C5mAb(Eculizumab)	3d	
–	Kidney transplantation	4KO (GGTA1, CMAH, B4GALNT2, GHR) + 6KI (hDAF, hCD46, hTBM, hEPCR, hCD47, hHO1)	Brain-dead	rATG + Anti-CD20mAb(Rituximab) + TAC + MMF + MP/Pred + Anti-C5mAb(Eculizumab)	7d	
Nov. 2022	Kidney transplantation	3KO (GGTA1, CMAH, B4GALNT2) + hDAF KI	Brain-dead	rATG + Anti-CD20mAb(Rituximab) + TAC + MMF + MP	12d	(8)
Jun.2023	Kidney transplantation	3KO (GGTA1, CMAH, B4GALNT2) + 2KI(hDAF, hTBM)	Brain-dead	rATG + Anti-CD20mAb(Rituximab) + TAC + MMF + MP + + Anti-C5mAb(Eculizumab)	12d	
Jan. 2022	Heart transplantation	4KO (GGTA1, CMAH, B4GALNT2, GHR) + 6KI (hDAF, hCD46, hTBM, hEPCR, hCD47, hHO1)	Living	rATG + Anti-CD40mAb(KPL-404) + MP + C1INH(Berinert) + Plasmapheresis + IVIG	60d	(10, 97)
Jun. 2022	Heart transplantation	4KO (GGTA1, CMAH, B4GALNT2, GHR) + 6KI (hCD59, hCD46, hTBM, hEPCR, hCD47, hHO1)	Brain-dead	rATG + MMF + MP + Anti-C5mAb(Eculizumab)	66h	(98, 99)
Jul. 2022	Heart transplantation	4KO (GGTA1, CMAH, B4GALNT2, GHR) + 6KI (hCD59, hCD46, hTBM, hEPCR, hCD47, hHO1)	Brain-dead	rATG + MMF + MP + Anti-C5mAb(Eculizumab)	66h	
Jul.2023	Thymokidney transplantation	GGTA1 KO	Brain-dead	rATG + Anti-CD20mAb(Rituximab) + CTLA4-Ig(Belatacept)+ TAC + MMF + MP/Pred + Anti-C5mAb(Eculizumab)/C3 inhibitor(Pegcetacoplan) + Plasmapheresis	61d	(26, 100)
Sep. 2023	Heart transplantation	4KO (GGTA1, CMAH, B4GALNT2, GHR) + 6KI (hDAF, hCD46, hTBM, hEPCR, hCD47, hHO1)	Living	Anti-CD154mAb(Tegoprubart)+ C1INH(Berinert)/Anti-C5mAb(Eculizumab)	40d	(101)
Mar. 2024	Heterotopic auxiliary liver transplant	3KO (GGTA1, CMAH, B4GALNT2) + 3KI (hDAF, hCD46+ hTBM)	Brain-dead	rATG + Anti-CD20mAb(Rituximab) + CTLA4-Ig(Etanercept) + TAC + MMF + MP + Anti-C5mAb(ravulizumab) + Plasmapheresis + IVIG	10d	(102, 103)
Mar. 2024	Kidney transplantation	3KO (GGTA1, CMAH, B4GALNT2) + 2KI (hDAF, hTBM)	Brain-dead	rATG + Anti-CD20mAb(Rituximab) + TAC + MMF + MP + Anti-C5mAb(Eculizumab) + Plasmapheresis + IVIG	22d	(104)
Mar. 2024	Kidney transplantation	3KO (GGTA1, CMAH, B4GALNT2) + 7KI (hDAF, hCD46, hTBM, hEPCR, hCD47, hTNFAIP3, hHO1) + PERV KO	Living	rATG + Anti-CD20mAb(Rituximab) + Anti-CD154mAb(Tegoprubart)+ TAC + MMF + Pred + Anti-C5mAb(Eculizumab)	52d	(105)
May. 2024	lung transplantatio	3KO (GGTA1, CMAH, B4GALNT2) + 3KI (hDAF, hCD46+ hTBM)	Brain-dead	rATG + Anti-CD20mAb(Rituximab) + Anti-CD25mAb(Basiliximab) + CTLA4-Ig(Belatacept) + TAC + MMF + MP + Anti-C5mAb(ravulizumab)	9d	(11)
Jan. 2025	Kidney transplantation	3KO (GGTA1, CMAH, B4GALNT2) + 7KI (hDAF, hCD46, hTBM, hEPCR, hCD47, hTNFAIP3, hHO1) + PERV KO	Living	–	271d	–
Mar. 2025	Kidney transplantation	3KO (GGTA1, CMAH, B4GALNT2) + 3KI (hDAF, hCD46+ hTBM)	Living	–	261d	–
May. 2025	Auxiliary partial orthotopic liver transplantation	3KO (GGTA1, CMAH, B4GALNT2) + 7KI (hDAF, hCD46, CD59, CD39, hTBM, hEPCR, hCD47)	Living	Anti-CD20mAb(Rituximab) + Anti-CD25mAb(Basiliximab) + TAC + Sirolimus+ MMF + MP + Anti-C5mAb(ravulizumab) + Plasmapheresis + IVIG	171d (38d remove graft)	(106)
Jun. 2025	Kidney transplantation	3KO (GGTA1, CMAH, B4GALNT2) + 7KI (hDAF, hCD46, hTBM, hEPCR, hCD47, hTNFAIP3, hHO1) + PERV KO	Living	–	>181d	–

### 6.1 Genetic strategy

Xenotransplantation faces significant challenges due to the stark antigenic differences between pig and human cells. These differences often lead to robust rejection reactions, both antibody-mediated and T-cell-mediated (107, 108). Knocking out (KO) surface carbohydrate antigens GGTA1, CMAH, and B4GALNT2 significantly reduces the pre-existing anti-pig antibodies in human and NHP sera, effectively lowering the incidence of antibody-mediated rejection (109–111). However, compared with WT pigs, GGTA1 knockout (GTKO) reduces human T cell proliferation in MLR, but there is no statistical difference compared with 3KO pigs (112, 113). Both GGTA1/CMAH/B4GALNT2/CIITA and GGTA1/B2M/CIITA knockout pigs exhibited reduced human T-cell proliferation and cytotoxic activation in MLR, indicating that down-regulation of SLA expression can alleviate the direct pathway response of T cells (37, 38). Because the SLA-I of pigs are difficult to interact with human CD94/NKG2A, the inhibitory signals of NK cells, CTLs and γδ T cells cannot be effectively triggered (41, 107, 114). This leads to an enhanced direct cytotoxic response against porcine cells. Introducing human HLA-E or HLA-G into porcine cells can partially restore these inhibitory pathways, thereby reducing the direct cytotoxicity of these cells (115–117). Meanwhile, downregulation of SLA expression in donor pigs can impair T-cell development and compromise antimicrobial immunity, thereby increasing susceptibility to infection and feeding under barrier conditions (37). Although current donor engineering has largely focused on deleting the major carbohydrate xenoantigens (GGTA1, CMAH and B4GALNT2) and modulating SLA expression (for example, via B2M and CIITA), additional xenoantigens remain potential immune targets, including diverse surface glycoproteins (such as β1 integrin and the transferrin receptor) and extracellular matrix (118, 119). Proteomic profiling of recipient serum reactivity to porcine proteins, together with HLA-I immunopeptidome analysis and epitope-presentation prediction, offers complementary, data-driven approaches to identify novel xenoantigens relevant to xenorecognition (119, 120). Phelps group constructed a porcine cytotoxic T-lymphocyte-associated protein 4 immunoglobulin (CTLA4-Ig), but pCTLA4-Ig had poor binding to human CD80/CD86 and caused fatal infections due to immunodeficiency (121, 122). Interestingly, pig PBMC expressing pCTLA4-Ig could reduce the immune response of hCD4+ T cells in co-culture, but compared with hCTLA4-Ig, the binding of pCTLA4-Ig to hCD80/CD86 was significantly weakened (123). Some groups constructed transgenic pigs expressing hCTLA4-Ig (Abatacept) or its affinity-optimized derivative LEA29Y (Belatacept), which permits sexual reproduction and extends the survival period of xenogeneic grafts in humanized mice and NHP models (124–127). Another similar method is to express hPD-L1 on pig cells, and through the PD-1/PD-L1 pathway, T cell activation can be continuously inhibited locally in the graft and apoptosis induced (128). *In vitro* experiments, the proliferation response of human CD4+ T cells in a co-culture system with hPD-1-expressing pig APC cells was significantly reduced, and the apoptosis of CD8+ T cells was significantly increased, promoting the expansion of CD25hiFoxp3+ Treg cells (128, 129). Overexpression of hFas in PECs induces marked apoptosis of human T cells and NK cells. Therefore, hFasL also constitutes a promising strategy to reduce cellular rejection in xenotransplantation (130). Notably, extensive genetic modification of donor pigs can be counterproductive, as it may compromise donor physiological fitness and graft viability while increasing the risk of off-target mutagenesis and genomic instability (131).

### 6.2 Induction and co-stimulation-based immunosuppression

In many NHP xenotransplantation models, immunosuppression protocols adapted from conventional allotransplant practice are associated with limited graft survival and substantial graft injury (132). Given this immunological characteristic, an induction protocol for T cell depletion is usually adopted, with anti-thymocyte globulin (ATG) being the most commonly used drug (20, 132). The preoperative use of ATG can usually maintain T cells at a low level within five weeks after surgery. In a porcine-NHP xenotransplantation model, the use of Anti-CD4Ab instead of Anti-CD8Ab also resulted in long-term survival, suggesting the importance of preoperative depletion of CD4+ T cells (83). In the study of porcine-human xenotransplantation, some groups also used Anti-CD25mAb (Basiliximab), which may inhibit T cell activation and proliferation by blocking the IL-2 pathway (11, 106). CTLA-4Ig can reduce the probability of cell rejection in allogeneic transplantation and induce immune tolerance (133–135). However, in multiple primate animal models, CD40-CD154 pathway blockade (anti-CD154 mAb or anti-CD40 mAb) has been associated with reduced graft T-cell infiltration compared with CTLA-4Ig-based regimens and with attenuated donor-specific antibody formation and antibody-mediated rejection (136, 137). In a 61-day clinical study of brain-dead patients, researchers found that in the immunosuppressive regimen based on CTLA-4Ig, there was a significant expansion of donor-reactive CD8+ T cells in the peripheral blood PBMC in the early stage of transplantation (26). Blocking the CD40-CD154 pathway in patients with acute cellular rejection of kidney transplantation can reverse the high oligoclonality of CD8+ T cells and significantly suppress the Tfh-related CD4+ T cells and B cell germinal center-like responses in the graft (138, 139). It is worth noting that the first-generation IgG1 anti-CD154 antibodies were repeatedly reported to cause thrombotic events in clinical trials, becoming one of the direct reasons for the termination of development. These antibodies can bind to FcγRIIa through their Fc fragment, mediate platelet activation and aggregation, thereby inducing drug-related thrombosis (127, 128). In this context, the new generation of anti-CD154 molecules reduce the risk of thrombosis by weakening the binding of Fc to FcγR. AT-1501/tegoprubart and other new anti-CD154 molecules currently show good tolerance in trials for amyotrophic lateral sclerosis and early transplantation-related trials, and no clear trend of thrombus aggregation has been found in the short-term safety data, but the follow-up time is limited and is not sufficient to completely negate its long-term risks (140, 141). Although Anti-CD154mAb and Anti-CD40mAb have not been approved for commercial use, they have been applied in multiple brain-dead donor models and individual xenotransplantations implemented under compassionate use. In *in vitro* experiments, Zhang found in MLR that at a low concentration of tacrolimus, the proliferation rate of T cells could be reduced to a minimal level, but rapamycin even at high concentrations only had a moderate inhibitory effect (142). In another *in vitro* study, Li found that pretreatment of PEC with rapamycin could almost completely eliminate its ability to induce T cell proliferation, and this inhibition could not be reversed by PD-1 blockade, suggesting that mTOR inhibition not only acts directly on T cells, but also reduces the quality and intensity of the xenogeneic T cell response by lowering the immunogenicity remodeling of the pig endothelium (143).

### 6.3 Mixed hematopoietic chimerism

Mixed haematopoietic chimerism is defined as the coexistence of donor- and recipient-derived haematopoietic cells in a recipient following transplantation of donor haematopoietic stem and progenitor cells. Across rodent models, large-animal studies and clinical experience, it has been associated with the induction of durable, donor-specific allograft tolerance (144). By mixing haematopoietic chimerism, donor-derived APCs can continuously migrate into the thymus, thereby enabling the newly generated T cells of the newborn to undergo negative selection with the donor antigens (145). Yang group discovered in a mouse model of porcine hematopoietic chimeras that the newly generated T cells of the recipients showed a significant decrease in response to donor proliferation, and could accept donor-derived skin grafts, while still having a reaction to third-party antigens, demonstrating typical donor-specific T cell tolerance (146). Receptor macrophages rapidly clear porcine hematopoietic progenitor cells, significantly hindering the formation of mixed chimeras. Transgenic expression of hCD47 inhibits phagocytosis by interacting with SIRPαNOD on murine macrophages, thereby significantly enhancing the engraftment of porcine hematopoietic cells *in vivo* (147). In NHPs, the Yamada group combined intra-bone bone marrow transplantation (IBBMTx) with non-myeloablative conditioning (bone marrow cell dose: 1.8–5.9 × 10^9^ cells/kg); this approach induced peripheral blood macrochimerism for up to 21 days and bone marrow engraftment detectable for up to 28 days (148, 149). In a baboon porcine lung transplantation study, hCD47-transgenic pigs used as both marrow and lung donors enabled durable macrochimerism exceeding 8 weeks with lower marrow cell doses (<1 × 10^9^ cells/kg) and prolonged lung xenograft survival (150). Notably, both IBBMTx studies reported reductions in anti-pig antibody titres and complement-dependent cytotoxicity, together with cellular hyporesponsiveness in MLR and IFNγ ELISPOT assays, consistent with attenuated anti-pig humoral and cellular immunity (148, 150).

### 6.4 Thymic transplantation

Transplanting donor pig thymus or vascularized thymic tissue to the recipient enables the T cell precursors of the recipient to undergo positive and negative selection in the pig thymus microenvironment (40). Previous studies on humanized mice have demonstrated that pig thymus can support the generation of human T cells with a diverse T cell receptor repertoire (151, 152). The T cells developed in pig thymus exhibit a specific low response to the donor pig, while retaining the ability to react to third-party pigs and human antigens (151, 153). In large animals, incomplete clearance of T cells may pose challenges in achieving tolerance during thymus transplantation (154). Thymus-plus-kidney grafts (Thymokidneys) can be obtained by performing partial thymectomy on pigs aged 6 to 8 weeks and transplanting autologous thymus tissue under the renal capsule (155). The combined transplantation of vascularized thymic grafts with kidneys enables the thymic tissue to be continuously perfused and participate in the generation of new T cells in the recipient’s body in a physiological manner (156). In recent attempts at pig-to-human kidney xenotransplantation involving brain-dead recipients and patients with end-stage renal failure, researchers have used thymokidneys obtained from GalT-KO pigs. Evidence of recipient thymic development within these grafts supports the feasibility of translating the thymic transplantation strategy from NHP models to the clinical pig-to-human setting (26).

### 6.5 Cell-based immunomodulatory strategy

Cell therapy based on immunomodulatory cells is gradually regarded as an important supplementary strategy for inducing functional tolerance and alleviating the maintenance of immunosuppression in xenotransplantation. Cynthia purified and expanded pig antigen-specific baboon CD4+CD25hi Treg cells *in vitro*, and compared with primary Treg cells, the expanded Treg cells could significantly inhibit T cell proliferation responses and cytokine secretion in MLR (157). CD27+ xeno-Tregs exhibit a more regulatory phenotype than CD27- counterparts, as evidenced by the upregulation of Foxp3, CTLA-4 and Helios, and the downregulation of IL-17. When these cells are administered to mice bearing porcine skin grafts, they maintain the normal morphology of the graft tissue with reduced leukocyte infiltration (158). In xenogeneic and cross-species hematopoietic stem cell transplantation-related models, Treg infusion was reported to be able to alleviate acute graft-versus-host disease to some extent and improve the quality of immune reconstitution (159). In NHP models, three rhesus monkeys received autologous polyclonal regulatory T-cell infusions after porcine islet xenotransplantation, and two of them maintained insulin independence for more than 500 days (160). MSCs have multi-directional differentiation potential and significant immunomodulatory and tissue repair capabilities. They can inhibit T and B cell activation through various soluble factors such as PGE_2_, TGF-β, IL-10, and cell contact, induce Treg expansion, and regulate the differentiation of dendritic cells to a tolerogenic phenotype (161). Pig MSCs can alleviate immunogenicity by knocking out GGTA and expressing human complement regulatory proteins, and can effectively down-regulate human T cells’ response to pig antigens, similar to human MSCs (162). Co-encapsulating pig islets with pig MSCs improved oxygenation and new angiogenesis in rat models and primate models (163). Besides Tregs and MSCs, other cell therapies, such as myeloid-derived suppressor cells (MDSCs) and regulatory macrophages (Mregs), are expected to form a multi-cellular synergistic regulatory network with Tregs and MSCs (164).

## 7 Conclusion

As xenotransplantation technology advances towards clinical application, T cells emerge as both a formidable barrier and a pivotal target for intervention, holding the key to unlocking the full potential of this groundbreaking field. Across pig-to-primate and pig-to-human xenotransplantation models, rejection is closely linked to vigorous T-cell activation, initiated through direct, indirect and semi-direct antigen-recognition pathways. In addition, expansion of donor-reactive T cells in both the periphery and the graft is thought to contribute to xenograft injury (26). Conventional immunosuppressive regimens struggle to achieve stable control of T cell-mediated xenotransplant rejection (132). Donor pig gene editing combined with co-stimulation blockade has fundamentally reshaped the field. Multigene editing markedly reduces graft immunogenicity, and when coupled with blockade of the CD40–CD154 or CD28–B7 pathways, has enabled prolonged xenograft survival in NHP models, with early clinical experience providing supportive evidence for these advances.

Future research should shift from broad T-cell suppression to precise, donor-specific control of xenoreactive T cells. Approaches such as durable mixed hematopoietic chimerism, thymic transplantation and regulatory cell-based therapies aim to promote tolerance by enabling selective deletion of donor-reactive clones and/or their functional regulation. Achieving safe, donor-directed control of T-cell immunity will be crucial for realizing the clinical potential of xenotransplantation.
